# Delayed pericarditis following ethanol ablation of the vein of Marshall in the treatment of atrial fibrillation: a case report

**DOI:** 10.11604/pamj.2024.47.20.42399

**Published:** 2024-01-18

**Authors:** Hao-Zhen Miao, Lei Tao, Bing-Jie Song, Yong Cao, Qi-Jun Zhang

**Affiliations:** 1 Department of Cardiology, the Affiliated People's Hospital of Ningbo University, Ningbo, China

**Keywords:** Delayed pericarditis, atrial fibrillation, catheter ablation, case report

## Abstract

In this case report, we will discuss a 74-year-old female who presented with a chief complaint of abdominal pain, bloating, anorexia, and nausea for four days which preceded after catheter ablation and anhydrous ethanol infusion vein of Marshall (VOM) one month prior. She was admitted and treated as a general patient in the general ward. After hospital admission, a pericardiocentesis was guided by B-scan ultrasonography, resulting in the extraction of 20ml of pericardial effusion, followed by catheterization for drainage. The key takeaway in this report is that anhydrous ethanol infusion VOM may not always be without risks. Hence, during the procedure, it is imperative to carefully administer the appropriate volume of anhydrous ethanol into the VOM to prevent vessel damage and associated complications.

## Introduction

The application of anhydrous ethanol injection into the VOM (EIM) during radiofrequency ablation for atrial fibrillation is a recent advancement in the field, offering a highly feasible approach for treating atrial tachyarrhythmia with a low rate of severe complications [1,2]. This technique employs X-ray fluoroscopy and angiography to chemically ablate the atrial muscle tissue within the VOM using anhydrous ethanol perfusion through the coronary sinus vein. It enhances the isolation of pulmonary vein potential, optimizes conditions for subsequent catheter radiofrequency ablation, minimizes postoperative leakages, and increases the success rate of isolating pulmonary vein potential during atrial fibrillation radiofrequency ablation, thereby reducing the occurrence of postoperative atrial tachycardia. However, anhydrous ethanol venous ablation carries a risk of complications, such as pericardial tamponade, and anaphylactic shock, among others. In this case report, we will describe a 74-year-old female who presented with a chief complaint of abdominal pain, bloating, anorexia, and nausea for four days which preceded after catheter ablation and anhydrous ethanol infusion VOM one month prior.

## Patient and observation

**Patient information:** we present a 74-year-old female patient with atrial fibrillation (AF) who presented with 4 days' history of abdominal pain, bloating, anorexia, and nausea. The symptoms started 1 month later after catheter ablation and anhydrous ethanol infusion VOM. No history of chest trauma, no loss of consciousness, and no change in micturition pattern. Other symptoms were unremarkable. She is a non-alcohol drinker and a non-smoker.

**Clinical findings:** upon physical examination, her vital signs were recorded as follows: body temperature 36.6°C, pulse rate 93 beats per minute, respiration rate 19 breaths per minute, and blood pressure at 133/76 mmHg. Further examination revealed an irregular heart rate at 95 beats per minute with no pathological murmurs detected, other vitals were stable.

**Timeline of the current episode:** the patient was referred and admitted to our unit on 30^th^ April 2021. catheter ablation and anhydrous ethanol infusion VOM was done on 5^th^ May 2021. The patient was readmitted on 20^th^ June 2021. A pericardiocentesis guided by B-scan ultrasonography was performed on the same day. Hospital discharge on 30^th^ June 2021.

**Figure 1:**
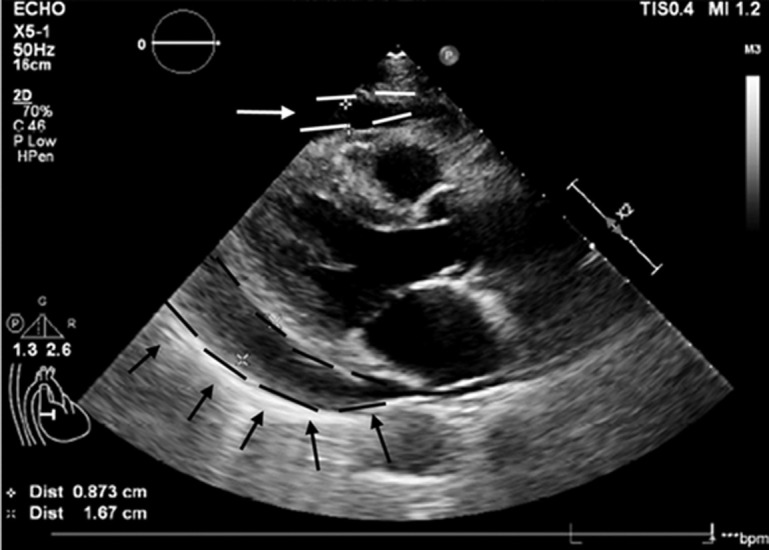
B-scan ultrasonography on admission showing massive bilateral pleural effusion

**Figure 2:**
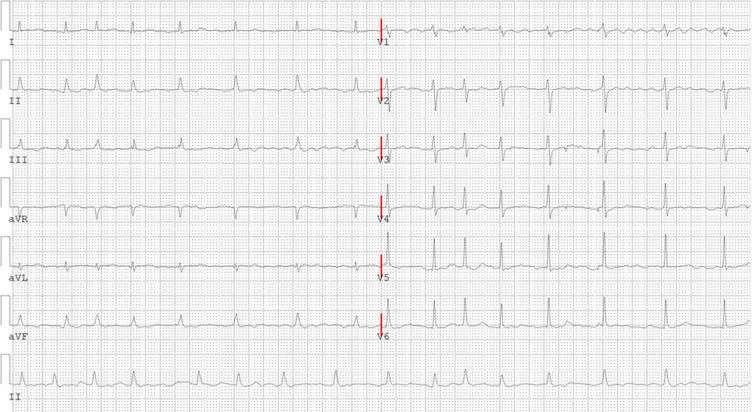
electrocardiogram on admission

**Diagnostic assessment:** cardiac ultrasound revealed specific measurements: left atrial diameter (end-systole) of 34mm, left ventricular diameter (end-diastole) of 43mm, left ventricular diameter (end-systole) of 26mm, interventricular septal thickness (end-diastole) of 8mm, left ventricular posterior wall thickness (end-diastole) of 8mm, and a left ventricular ejection fraction of 65%. Ultrasound indicated a reduction in left ventricular diastolic function. Pericardial effusion (Figure 1) and ECG (Figure 2) was observed. Echocardiography of the pericardium revealed fluid separation in the anterior pericardium and apex, measuring approximately 26mm in width.

**Diagnosis:** delayed pericarditis; atrial fibrillation.

**Therapeutic interventions:** after hospital admission, a pericardiocentesis was guided by B-scan ultrasonography, resulting in the extraction of 20ml of pericardial effusion, followed by catheterization for drainage. Twenty-four hours of post-surgery, echocardiography of the pericardial cavity indicated an absence of fluid accumulation. Consequently, the drainage tube was removed, and the patient was discharged. The pericardial effusion obtained was hemorrhagic, the biochemical results were suggestive of normalcy and the Levanta test was weakly positive.

**Follow-up and outcomes:** in the postoperative follow-up, the patient did not report any notable discomfort.

**Patient perspective:** the patient was delighted with the quality of care.

**Informed consent:** written informed consent was obtained from the patient for the publication of this case report.

## Discussion

**Figure 3:**
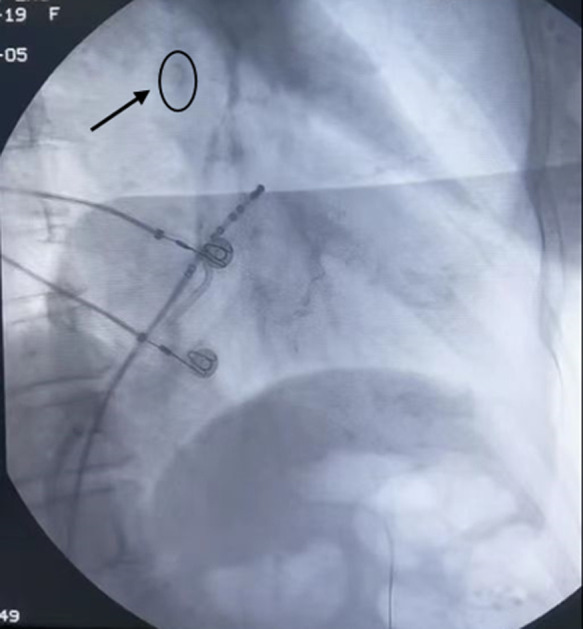
the black arrow indicates the anhydrous ethanol and contrast media in the surgery video

Currently, radiofrequency ablation involving ethanol injection into blood vessels is widely employed in clinical practice. It is utilized in conditions like mitral flutter [3] and peri-mitral reentrant tachycardia [4], as well as in certain cardiomyopathies, including hypertrophic obstructive cardiomyopathy [5]. Nonetheless, anhydrous ethanol venous ablation carries a risk of complications, including pericardial tamponade [6], and anaphylactic shock, among others. The primary factors contributing to these complications are excessive force and vagal reflex during the ablation procedure. Furthermore, research has indicated that being female can be an independent risk factor for pericardial effusion following radiofrequency ablation [7]. Nonetheless, a recent meta-analysis suggests that anhydrous ethanol intravenous ablation is generally considered safe and dependable [8]. In our case, the patient experienced pericardial tamponade one month after EIM. Upon reviewing the surgical video of this patient, we determined that during the procedure, anhydrous ethanol and contrast media damaged the VOM, leading to the development of pericardial effusion post-surgery (Figure 3). Additionally, one potential mechanism for delayed pericardial effusion following EIM may involve the impact of anhydrous alcohol and pericardial injury, leading to inflammation [9].

A different study related to EIM indicates that injecting 4mL of anhydrous ethanol into the human VOM is generally considered safe [10]. Nonetheless, in this case, 12ml of anhydrous ethanol was administered during the procedure, leading to the development of pericardial effusion in the patient a month after the surgery. This incident serves as a reminder that EIM may not always be without risks.

## Conclusion

Hence, it is imperative to carefully administer the appropriate volume of anhydrous ethanol into the VOM to prevent vessel damage and associated complications during the procedure.
